# miRgo: integrating various off-the-shelf tools for identification of microRNA–target interactions by heterogeneous features and a novel evaluation indicator

**DOI:** 10.1038/s41598-020-58336-5

**Published:** 2020-01-30

**Authors:** Yen-Wei Chu, Kai-Po Chang, Chi-Wei Chen, Yu-Tai Liang, Zhi Thong Soh, Li‐Ching Hsieh

**Affiliations:** 1 Institute of Genomics and Bioinformatics, National Chung Hsing University, Taichung, 402 Taiwan; 2 Agricultural Biotechnology Center, National Chung Hsing University, Taichung, 402 Taiwan; 3 Institute of Molecular Biology, National Chung Hsing University, Taichung, 402 Taiwan; 4 Biotechnology Center, National Chung Hsing University, Taichung, 402 Taiwan; 5 Rong Hsing Research Center For Translational Medicine, National Chung Hsing University, Taichung, 402 Taiwan; 6 Ph.D. Program in Medical Biotechnology, National Chung Hsing University, Taichung, 402 Taiwan; 7 China Medical University Hospital, Taichung, 404 Taiwan; 8 Department of Computer Science and Engineering, National Chung Hsing University, Taichung, 402 Taiwan; 9 Department of Life Science, National Chung Hsing University, Taichung, 402 Taiwan; 10 Advanced Plant Biotechnology Center, National Chung Hsing University, Taichung, 402 Taiwan; 11 Department of Physics, National Chung Hsing University, Taichung, 402 Taiwan

**Keywords:** Computational models, Computational platforms and environments, Data integration, Gene regulatory networks, Machine learning

## Abstract

MicroRNAs (miRNAs) are short non-coding RNAs that regulate gene expression and biological processes through binding to messenger RNAs. Predicting the relationship between miRNAs and their targets is crucial for research and clinical applications. Many tools have been developed to predict miRNA–target interactions, but variable results among the different prediction tools have caused confusion for users. To solve this problem, we developed miRgo, an application that integrates many of these tools. To train the prediction model, extreme values and median values from four different data combinations, which were obtained via an energy distribution function, were used to find the most representative dataset. Support vector machines were used to integrate 11 prediction tools, and numerous feature types used in these tools were classified into six categories—binding energy, scoring function, evolution evidence, binding type, sequence property, and structure—to simplify feature selection. In addition, a novel evaluation indicator, the Chu-Hsieh-Liang (CHL) index, was developed to improve the prediction power in positive data for feature selection. miRgo achieved better results than all other prediction tools in evaluation by an independent testing set and by its subset of functionally important genes. The tool is available at http://predictor.nchu.edu.tw/miRgo.

## Introduction

MicroRNAs (miRNAs) are short non-coding RNAs (~21 nucleotides) that have important roles in cell biology. miRNAs are involved in the control of a variety of physiological processes including development, cell proliferation, apoptosis, tissue differentiation and metabolism, by binding to and then silencing translation of target mRNAs^1–4^. The function of miRNAs in regulation of gene expression was first described by researchers studying *C*. *elegans*; they found that miRNA *lin-4* was able to suppress the expression of the *lin-*14 target gene^5^. In animals, the mechanism by which miRNAs silence gene expression can be described in three steps. In the first step, a hairpin-shaped transcript of the DNA encoding the miRNA, referred to as the primary miRNA, is trimmed by Drosha and Pasha into a loop-shaped structure ~70 nucleotides in length, resulting in the pre-miRNA. The pre-miRNA is then transported into the cytoplasm by exportin-5 and then is processed by Dicer to cleave the hairpin structure into two single strands. One of the strands becomes the mature miRNA and then binds with Argonaute protein to form an RNA-induced silencing complex, which blocks mRNA translation or induces mRNA degradation^6^.

To understand the function of a miRNA, one must first determine its target genes and binding sites, but this task can be challenging because miRNA–mRNA binding is often incomplete *in vivo*^7^, and the mechanism by which miRNAs are targeted to specific genes is mostly unknown. Most miRNAs bind to the 3′-untranslated region (UTR) of the target mRNA at a 2- to 8-nucleotide region near the 5′ end, the “seed region”^8^. Because there are often mismatches, gaps, and G:C wobble outside the seed region, it may be possible to deduce the binding site by identifying the seed region based on these features. However, G:C wobble often occurs in the seed region, so this approach is unreliable^9^. To study the miRNA–mRNA relationship, numerous laboratory methods, such as western blotting, luciferase reporter assay, green fluorescent protein (GFP) reporter assay, reverse transcription polymerase chain reaction (RT-PCR), pulsed stable isotope labeling by amino acids in cell culture (pulsed SILAC or pSILAC), microarray analysis, branched DNA probe assay, and northern blotting, have been used by researchers, but these laboratory methods often require considerable resources^10^. To save time and resources, many researchers have developed tools that predict miRNA–mRNA binding sites and the gene regulatory effect of miRNAs^11^.

Previously published miRNA target site prediction tools can be classified into five categories: sequence-based tools such as TargetScan^12^, miRanda^13^, PITA^14^, and PACCMIT-CDS^15^; energy-based tools such as PicTar^16^, RNAhybrid^17^, RNAduplex^18^, and microT-CDS^19^; machine learning–based tools such as MBSTAR^20^, MiRTDL^21^, TarPmiR^22^, and miRDB^23^; statistics-based tools such as RNA22^24^; and database-based tools such as StarMirDB^25^. The results generated by different tools are not always consistent. In other research fields that have problems in result variability among different tools, researchers often combine multiple tools into integrated systems. This approach has proven successful for predicting protein interactions^26^, protein subcellular location^27^, miRNA in transcripts^28^, and protein stability changes^29^. In this study, we have integrated existing tools to develop a novel prediction system, miRgo, which is free for use by researchers worldwide. This system integrates 11 prediction tools and was trained using miRTarBase^30^, which is a curated database of miRNA–mRNA interactions with 360,000 laboratory data entries obtained from western blotting, luciferase reporter assays, microarray analyses, and next-generation sequencing.

miRgo was developed with the support vector machine (SVM) algorithm^31^ and the minimum redundancy–maximum relevance (mRMR) feature selection method^32^. To reduce the size and dimension of the training set, three smaller datasets were obtained by filtering according to extreme values and middle values of binding energy, which was calculated with an energy distribution function. Another dataset, which was randomly selected from the database, was included for comparison. To reduce the number of features, the prediction results from different prediction tools were classified into six categories—binding energy, scoring function, evolution evidence, binding type, sequence property, and structure—to simplify the feature selection process. miRgo shows superior accuracy compared with other tools in 10-fold cross-validation^33^ of the top 30% of features of the training set. For testing with an independent testing set, because of the limited performance of Matthews Correlation Coefficient (MCC), Accuracy (Acc), and the F1 scores in interpretation of test results, an evaluation function, the CHL index, was designed and defined as the normalized harmonic mean of MCC, Acc, and the F1 score. This index prevents the accuracy paradox^34^ problem and emphasizes prediction of positive data. When evaluated by the CHL index and the F1 score, miRgo performed better than all other tools in the independent testing set and in its subsets of functionally important genes. A website tool based on miRgo was built, and prediction data generated using miRgo are reported for future use by researchers.

## Material and Methods

### Data collection and positive and negative set construction

There were 2,588 human mature miRNA sequences in miRBase^35^ version V21. We acquired 322,352 records describing the relationship between the 2,588 human miRNAs and 14,886 targets from miRTarBase release 7.0^30^. To reduce the amount and dimension of the data, the CD-HIT-EST^36^ clustering tool from the CD-HIT toolkit was used under a sequence identity threshold of 0.8. After removal of sequence redundancy, 292,686 records related to the 2,588 miRNAs were obtained and defined as the total positive dataset. The total negative dataset was generated by the permutation method described by Zhang *et al*.^37^.

**Figure 1:**
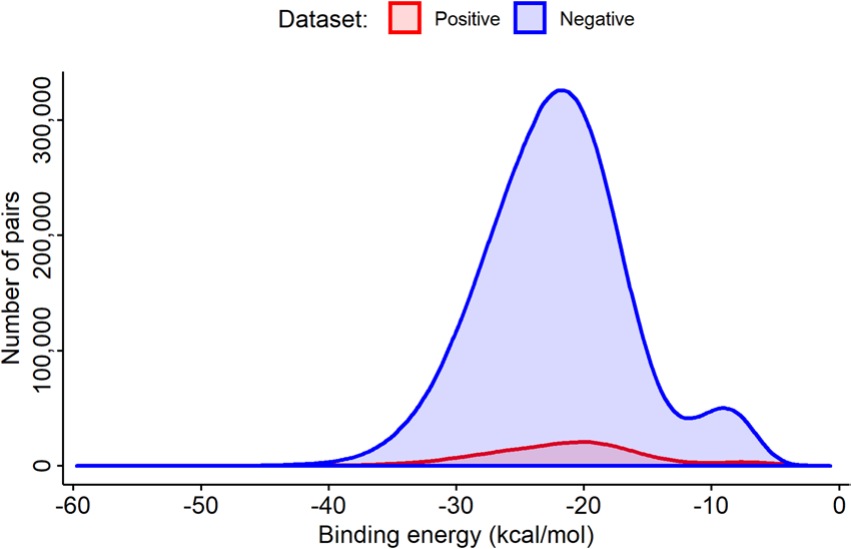
Distribution of binding energy of miRNA–mRNA pairs for positive (red) and negative data (blue).

To train the models, four training sets—trA, trB, trC, and trR—were used. The first three training sets (trA, trB, and trC) were selected from the aforementioned records using the binding energy distribution function (Fig. 1) obtained by RNAhybrid^17^ and RNAduplex^18^. The proportion of positive and negative data in the training sets was adjusted by a positive/negative (P/N) ratio analysis from the energy distribution function. The trA dataset, with 5,176 total records, consisted of the positive subset trA_P, which contained 2,588 records from the most stable miRNA–target pairs to the 2,588 selected miRNAs, and the negative subset trA_N, which contained 2,588 records from the most unstable pairs. The trB dataset, with 10,352 total records, consisted of 5,176 records from the most stable and second-most stable pairs (the trB_P subset) and 5,176 records from the most unstable pairs and second-most unstable pairs (the trB_N subset). The trC dataset, with 10,352 total records, consisted of pairs with extreme and mid-range binding energy. The last training set, trR, consisted of 10,532 randomly selected records. To test the models, 1,877 data records related to 38 miRNAs and 1,258 genes retrieved from MiRTDL^21^ (originally from TarBase v7.0^38^) were used. After removal of data that was duplicated in the training sets, the testing data included 1,248 positive records and 241 negative reports. In addition, the genes with the Gene Ontology^39^ annotation in the aforementioned testing set were selected for evaluation of the accuracy of the models.

### Tool integration

Since 2005, a number of computational tools for predicting miRNA–target interactions have been published (Table 1). To build the miRgo prediction system, a meta-predictor was developed via integration by SVM of 11 of the 14 prediction tools: RNA22, RNAhybrid, TargetScan, PITA, miRanda, RNAduplex, microT-CDS, StarMirDB, PACCMIT-CDS, MBSTAR, and TarPmiR. PicTar was excluded from integration because of its outdated database, miRDB was excluded because of the lack of information it provided, and information from MiRTDL was not integrated because it was used as a testing set for miRgo.

**Table 1:** Computational tools for predicting miRNA–target interactions.

Tool	Input type^a^	Method	Availability	Year	Integration^b^
PicTar	m or g	Sequence complementarity, thermodynamics and statistical model	Web-based	2005	x
RNA22^c^	m and g	Sequence complementarity, thermodynamics and statistical model	Stand-alone	2006	○
RNAhybrid^c^	m and g	Thermodynamics and statistical model	Stand-alone	2006	●
TargetScan	m and g	Sequence complementarity, thermodynamics	Web-based	2007	●
PITA^c^	m and g	Site accessibility, thermodynamics	Stand-alone	2007	○
miRanda^c^	m and g	Sequence complementarity, thermodynamics	Stand-alone	2008	●
miRDB	m or g	Machine learning (support vector machines)	Web-based	2008	x
RNAduplex	m and g	Thermodynamics and statistical model	Stand-alone	2011	●
microT-CDS^c^	m or g	Sequence complementarity, thermodynamics	Stand-alone	2012	●
STarMirDB	m and g	Sequence complementarity, thermodynamics and statistical model	Web-based	2013	●
PACCMIT-CDS	m and g	Sequence complementarity and statistical model	Web-based	2013	○
MBSTAR	m and g	Machine learning (support vector machines)	Stand-alone	2013	○
MiRTDL	m and g	Machine learning (convolutional neural network)	Web-based	2016	x
TarPmiR	m and g	Machine learning (random forest)	Stand-alone	2016	○

^a^The required input information. m: microRNA, g: gene.

^b^Whether the tool integrated in miRgo. ○: the tool integrated in miRgo, ●: the integrated tool with selected features, x: the tool not integrated in miRgo.

^c^These tools provide web-based service as well, but miRgo utilizes the results generated from stand-alone programs.

### Feature extraction and encoding

To integrate the results of the prediction tools, the feature encoding system must first be integrated. There are differences in feature encoding among the results of the prediction tools. The results of some prediction tools are encoded as 1 and 0 to represent a binding pair and a non-binding pair, respectively. The prediction results of miRanda^13^ are encoded into four categories: good mirSVR^13^ score, conserved miRNA (miRanda_S_C); good mirSVR score, non-conserved miRNA (miRanda_S_0); non-good mirSVR score, conserved miRNA (miRanda_0_C); and non-good mirSVR score, non-conserved miRNA (miRanda_0_0). The prediction results of STarMirDB^25^ are encoded into six categories: 3′ UTR-seed sites (STMDB_3US), 3′ UTR-seedless sites (STMDB_3ULS), CDS-seed sites (STMDB_CS), CDS-seedless sites (STMDB_CLS), 5′ UTR-seed sites (STMDB_5US), and 5′ UTR-seedless sites (STMDB_5ULS). To develop miRgo, 32 feature types from the various tool results were selected for encoding and integrated into six categories: energy, scoring function, evolution evidence, binding type, sequence property, and structure. The feature types selected are listed in Table 2 and are explained below. All feature encoding systems included are listed in Supplementary Table S1.

**Table 2:** The features utilized in miRgo.

Feature category	Feature^a^
Energy	binding energy, minimum free energy, folding energy
Scoring function	mirSVR score, context score, RNA22 p-value, RNAhybrid p-value, logistic probability of the site, miTG score, PACCMIT-CDS p-value, binding probability, m/e motif
Evolution evidence	conservation, Pct
Binding type	gene start and end sites, microRNA start and end sites, seed type, binding position, binding site, seed match
Sequence property	alignment score, nucleotide composition, AU content
Structure	ΔG_hybrid_, ΔG_nucl_, ΔG_total_, ΔG_duplex_, ΔG_open_, ΔΔG, accessibility

^a^The description for each feature is listed in Supplementary Table S1.

The seed type of the miRNA–gene binding based on the results of the prediction tools STarMirDB^25^, PITA^14^, MBSTAR^20^, and TargetScan^12^ was taken as an encoded feature. For encoding of the seven canonical seed types used in these tools, a seven-dimension vector was constructed. If a particular seed type was present in a miRNA–target pair, the value of that seed in the vector was set to 1; otherwise the value was set to 0. The feature codes are shown in Supplementary Table S2.

The dataset in TargetScan includes binding position and range, which can be encoded into the nucleotide composition of the binding site sequence. In addition, each record from the prediction results of miRanda, RNAduplex, and StarMirDB, which includes information about the starting and ending positions of a binding site, can also be converted into the nucleotide composition of the binding site sequence. No range information is included in PITA and MBSTAR, so only the binding position was obtained and encoded as data from these tools. For miRNA–target pairs with no prediction result, the value for the position and nucleotide proportion was set to 0.

### Feature selection and model construction

**Figure 2:**
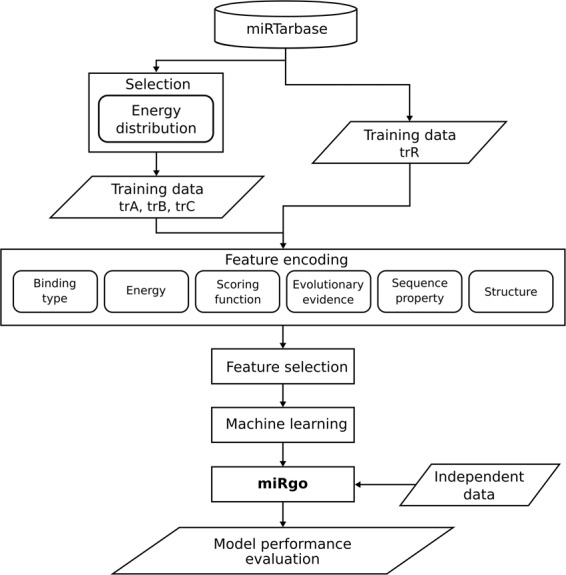
Flowchart of the miRgo prediction system.

After constructing the training sets and integrating the prediction methods and feature encoding system (as described above, we selected the dataset used for training of the classifiers based on 10-fold cross-validation. To further improve the accuracy of the classifiers, a number of features selected by the incremental feature selection (IFS) method^40^ followed by the mutual information quotient (MIQ) scheme of the mRMR method^32^ were included in the model. The SVM classifier and learning method were selected by comparing seven classifiers from the Weka toolkit^41^—baye, function, lazy, meta, misc, rule, and tree—and 47 learning methods with LIBSVM^31^. Because of the difficulty in obtaining consistent results using current evaluation indicators to evaluate the performance of miRgo and other tools, a novel evaluation indicator, the CHL index, was developed and is described in next paragraph. The miRgo development flowchart is shown in Fig. 2.

### Classifier performance evaluation

To evaluate the performance of classifiers, four values are commonly measured: (1) true positive rate (TP), the proportion of miRNA-target pairs that bind to each other and are correctly predicted by the classifier as binding pairs; (2) false negative rate (FN), the proportion of pairs that bind to each other but are falsely predicted by the classifier as non-binding pairs; (3) false positive rate (FP), the proportion of pairs that do not bind to each other but are falsely predicted by the classifier as binding pairs; and (4) true negative rate (TN), the proportion of pairs that do not bind to each other and are correctly predicted by the classifier as non-binding pairs. Several evaluation metrics—accuracy (Acc), sensitivity (Sn), specificity (Sp), precision, the F_1_ score, and Matthews correlation coefficient (MCC)—can be obtained from TP, FN, FP, and TN. These metrics are shown in Formulas 1–6.

Acc, an indicator of overall prediction accuracy, is calculated as shown in Formula 1.

1 $$Acc=\frac{TP+TN}{TP+TN+FP+FN}$$

Sn (also called recall) is an indicator of the power for detecting positives and is shown in Formula 2.

2 $$Sn=\frac{TP}{TP+FN}$$

Sp is an indicator of the power for detecting negatives and is shown in Formula 3.

3 $$Sp=\frac{TN}{TN+FP}$$

Precision is an indicator of the accuracy of predicting positives, as shown in Formula 4.

4 $$Precision=\frac{TP}{TP+FP}$$

The F_1_ score, or the F-measure, is a weighted arithmetic mean of precision and Sn. The range of this score is from 0 to 1. It indicates the prediction accuracy for positive data. The F_1_ score is shown in Formula 5.

5 $${F}_{1}\,score=2\times \frac{Precision\times Sn}{Precision+Sn}$$

The MCC is an objective indicator that is used to evaluate prediction power on positives or negatives. By balancing the effect of positive and negative prediction accuracy, it is generally more reliable than Sn, Sp, or precision. The range of MCC is from −1 to 1. If MCC is equal to 1, the prediction is totally correct, and if MCC is equal to −1, the prediction is totally incorrect. All-positive or all-negative prediction will yield a MCC of 0. MCC is shown in Formula 6.

6 $$MCC=\frac{(TP\times TN)-(FP\times FN)}{\sqrt{(TP+FP)(TP+FN)(TN+FP)(TN+FN)}}$$

Among the prediction metrics described above, Acc is seemingly a useful indicator for accuracy, but its usefulness is actually limited because of the accuracy paradox, which also affects the F1 score^42^. Using MCC avoids the accuracy paradox, but because the tools that are focused solely on negative data prediction may still achieve a high MCC score, it is unreliable when positive data prediction is important. During construction of miRgo, we found that Acc, F1-score, and MCC were inconsistent when various models were compared. To avoid the pitfalls of these three metrics and to resolve these inconsistencies, we developed a metric, the CHL index, that represents the harmonic mean of the Acc, the F1 score, and MCC′ (a normalized MCC that has a value range of 0–1). The effect of positive and negative prediction data on the CHL index is between that of the F1 score and MCC, so positive prediction data will have more weight on the CHL index than in the MCC, and negative prediction data will have more weight on the CHL index than in the F1 score. The calculations for MCC′ and the CHL index are shown in Formulas 7 and 8.

7 $$MCC^{\prime} =\frac{MCC+1}{2}$$

8 $$The\,CHL\,index=3\times \frac{Acc\times MCC^{\prime} \times {F}_{1}}{(Acc\times MCC^{\prime} )+(MCC^{\prime} \times {F}_{1})+({F}_{1}\times Acc)}$$

## Results

### The positive-negative ratio optimization for training data

**Figure 3:**
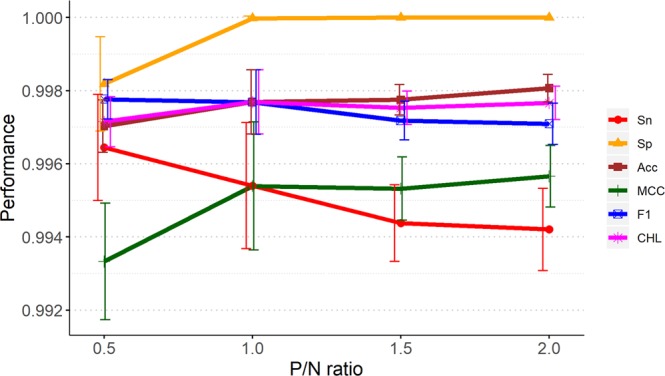
Model performance for various training sets based on different P/N ratios. For each P/N ratio, ten randomly sampled training sets were generated for performance evaluation using six indicators, Sn, Sp, Acc, MCC, the F1-score and the CHL-index.

An imbalance between positive and negative data may cause bias in machine learning. To search for an optimal P/N ratio for the training set, we designed models trained with four different P/N ratios. Figure 3 shows that when evaluated by the CHL index, a P/N ratio of 1:1 achieved the best result. The positive data were based on the 2,588 miRNA–target pairs. The negative data were generated by permutation and combination. The models were trained by 10 consecutive runs of SVM with randomly selected data sets.

### Classifier selection

After selecting the best training sets with a P/N ratio of 1:1, various classifiers were tested with the selected sets for accuracy. Five classifiers from the Weka toolkit, including baye, function, lazy, meta, and tree, and seven algorithms were tested and compared with LIBSVM. The results are shown in Supplementary Table S3.

### Training data for cross-validation

To select the best data set for model construction, data sets trA, trB, trC, and trR were tested with 10-fold cross-validation with a selected subset as the training set and other subsets as the validation or testing set. The results of trA, the best-performing data set, are shown in Table 3. The results of the other data sets are shown in Supplementary Tables S4–S6.

**Table 3:** Performance comparison of different miRNA–target interaction prediction methods for the trA set.

Prediction method	Sn	Sp	Acc	F_1_-score	MCC	MCC′	CHL-index
miRgo_trA^a^	0.9992	0.9981	0.9986	0.9986	0.9973	0.9986	0.9986
RNA22	0.7608	0.8798	0.8203	0.8090	0.6452	0.8226	0.8172
miRanda_0_0	0.1967	0.9691	0.5828	0.3204	0.2610	0.6305	0.4671
miRanda_0_C	0.0568	0.9903	0.5235	0.1065	0.1315	0.5657	0.2296
miRanda_S_0	0.1298	0.9896	0.5596	0.2277	0.2337	0.6169	0.3846
miRanda_S_C	0.0479	0.9934	0.5206	0.0909	0.1270	0.5635	0.2041
STMDB_3US	0.2434	1.0000	0.6216	0.3915	0.3722	0.6861	0.5338
STMDB_3ULS	0.4552	0.7248	0.5900	0.5261	0.1869	0.5934	0.5681
STMDB_CS	0.0000	1.0000	0.4999	null^b^	0.0000	0.5000	null^b^
STMDB_CLS	0.4892	0.5226	0.5059	0.4975	0.0118	0.5059	0.5031
STMDB_5US	0.0228	1.0000	0.5113	0.0446	0.1074	0.5537	0.1145
STMDB_5ULS	0.4451	0.4863	0.4657	0.4545	−0.0686	0.4657	0.4619
TargetScan	0.9668	0.9084	0.9376	0.9394	0.8767	0.9383	0.9384
DIANA_microT	0.2832	0.9988	0.6410	0.4410	0.4038	0.7019	0.5712
PITA	0.0978	0.9888	0.5432	0.1763	0.1906	0.5953	0.3263
TarPmiR	0.9610	0.9157	0.9384	0.9397	0.8776	0.9388	0.9390
MBSTAR	0.2902	0.7101	0.5001	0.3673	0.0003	0.5002	0.4463
PACCMIT-CDS	0.0761	0.9992	0.5376	0.1414	0.1959	0.5980	0.2829

^a^The miRgo_TrA model was trained on the trA training data with 10-fold cross validation.

^b^null: The F1-score and the CHL-index cannot be calculated because both TP and FP are zeros in this case.

When tested with trA, the prediction model miRgo–trA achieved the best results for all metrics among 18 tools (Table 3). Of the metrics tested, Sn showed the most variation among tools, and Sp showed the least variation. MCC also showed marked variation. The variation in the CHL index among the tools was less than that for MCC but was greater than that for Sp. When tested with trB, the prediction model miRgo–trB achieved the best results for all metrics among 18 tools, but it was less accurate than miRgo–trA (Supplementary Table S4). Its worse performance might be because some miRNAs have just one target (i.e. these miRNAs don’t have the second-most stable pair), so other miRNAs’ the third-most stable binding pair, which may have weaker binding energy, will be used instead, or because some second-most stable pairs had weaker binding energy. When tested with trC, the prediction model miRgo–trC performed better than most tools but showed a worse Acc, F1-score, and MCC than did TargetScan and TarPmiR, the two tools that may be more focused on mid-range data (Supplementary Table S5). When tested with trR, the prediction model miRgo–trR achieved the best results for all metrics among 18 tools but was still worse than miRgo–trA (Supplementary Table S6).

The best data set, trA, was used for final model training. To assess the characteristics of accuracy metrics, correlation between Acc, the F1 score, MCC′, and the CHL index was measured by testing with trA and various tools (Supplementary Fig. S1). Acc was closely correlated with MCC′ and was markedly different than the F1 score; the CHL index was closely correlated with the F1 score but was markedly different than Acc and MCC′. Thus, the CHL index may give more weight to negative data prediction power.

### Feature selection

To select a suitable feature selection method, we first compared the performance of six feature selection methods from the Weka^41^ toolkit—CVAttributeEval, GainRatioAttributeEval, InfoGainAttributeEval, OneRAttributeEval, CorrelationAttributeEval, and SymmetricalUncertAttributeEval—on model construction by trA. Because there was no meaningful performance difference, CVAttributeEval was arbitrarily selected to represent the Weka method. CVAttributeEval was then compared with the mRMR feature selection method for performance based on the incremental feature selection (IFS) procedure^40^. The performance of the method without feature selection, miRgo_trA, and the two feature selection methods, miRgo_trA_FS-mRMR and miRgo_trA_FS-CVAE, is shown in Table 4.

**Table 4:** Performance comparison of the miRgo models with and without the feature selection (FS) procedure for the trA set.

Model	Sn	Sp	Acc	F_1_-score	MCC	MCC′	CHL-index
miRgo_trA^a^	0.9992	0.9981	0.9986	0.9986	0.9973	0.9986	0.9986
miRgo_trA_FS-mRMR^b^	1.0000	0.9981	0.9990	0.9990	0.9981	0.9990	0.9990
miRgo_trA_FS-CVAE^b^	1.0000	0.9977	0.9988	0.9988	0.9977	0.9988	0.9988

^a^miRgo_TrA doesn’t include the feature selection (FS) procedure.

^b^miRgo_trA_FS-mRMR and miRgo_trA_FS-CVAEis are with the mRMR and CVAttributeEval feature selection method, respectively.

**Figure 4:**
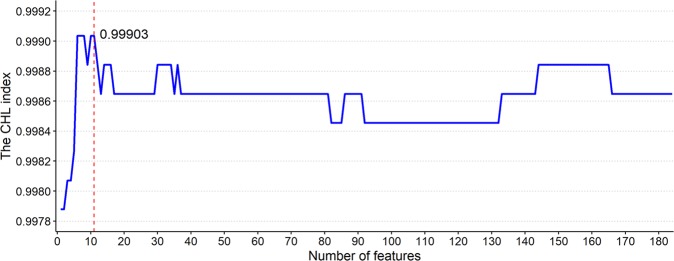
The incremental feature selection (IFS) curve of the combination of features. Features ranked by the mRMR method were added one by one from higher to lower rank into models, and 184 models with different combination of features were constructed and evaluated by the CHL index. It can be observed that the combination of 11 most important features makes the CHL index to reach a maximum value of 0.99903.

The model trained by the trA set without feature selection scored 0.9990, 0.9973, and 0.9986 on Sn, MCC, and the CHL index, respectively. After the CVAttributeEval feature selection was conducted, Sn, MCC, and the CHL index increased to 1.0000, 0.9977, and 0.9988, respectively. After the mRMR feature selection was conducted, Sn, MCC, and the CHL index increased to 1.0000, 0.9981, and 0.9990, respectively. Because of better performance, the MIQ scheme of the mRMR method was chosen for model construction. Based on the ranked features evaluated by the mRMR method, the IFS procedure was then used to determine the optimal number of features. During the IFS procedure, features in the ranked feature list are added one by one from higher to lower rank, and then 184 different feature subsets are obtained. An IFS curve, revealing the relation between the CHL index and the feature subset, is plotted in Fig. 4, which shows that several subsets with no more than eleven most important features would make the CHL index to reach maximum. We then chose the eleven features from six tools, including minimal free energy, predicted binding position, and p-value from RNAhybrid; context score and seed type from TargetScan; nucleotide proportion from miRanda(A,C) and RNAduplex; endpoints of the predicted binding site from StarMirDB, p-value from RNA22, and miTG score from DIANA-microT for model construction. These features cover energy, scoring function, binding type, and sequence property.

To examine whether overfitting occurred, independent testing was conducted on models built before and after feature selection. The test showed that miRgo_trA_FS-CVAE scored better than miRgo_trA_FS-mRMR for Sn, A, and the F1 score but scored worse than miRgo_trA_FS-mRMR for MCC and the CHL index, the two metrics that consider both Sn and Sp. miRgo_trA_FS-mRMR was superior if positive and negative data were concerned, and the CHL index enhanced the importance of positive data while retaining the accuracy paradox−solving ability of MCC (Table 5).

**Table 5:** Performance comparison of the miRgo models with and without the feature selection (FS) procedure for the independent test set.

Model	Sn	Sp	Acc	F_1_-score	MCC	MCC′	CHL-index
miRgo_trA	0.7765	0.4066	0.7180	0.8226	0.1538	0.5769	0.6910
miRgo_trA_FS-mRMR	0.8840	0.2900	0.7900	0.8760	0.1810	0.5905	0.7316
miRgo_trA_FS-CVAE	0.9354	0.1411	0.8098	0.8923	0.1480	0.5524	0.7201

### Model evaluation by independent testing dataset

To compare the performance of miRgo_trA_FS-mRMR with other prediction tools, testing was conducted with an independent dataset. miRgo_trA_FS-mRMR performed better than all other tools when measured by Acc, the F1 score, and the CHL index. The only metric for which miRgo_trA_FS-mRMR performed worse than any tool was MCC, where MBSTAR yielded a score of 0.2807 and miRgo_trA_FS-mRMR yielded a score of 0.1810. The reason for the high MCC score of MBSTAR may be caused by low sensitivity, with only 451 records generated when tested with 1,525 records of independent data. By missing 833 records of positive data and 241 records of negative data, measuring MCC with MBSTAR may have falsely overestimated the accuracy based on negative data. Because most predictions of miRNA–target interactions focus on positive data, the better measure would be the CHL index, which avoids the accuracy paradox while still focusing on positive data; miRgo_trA_FS-mRMR and MBSTAR scored 0.7316 and 0.5273, respectively. The independent testing results are shown in Table 6.

**Table 6:** Performance comparison of different miRNA–target interaction prediction methods for the independent test set.

Prediction method	Sn	Sp	Acc	F_1_-score	MCC	MCC′	CHL-index
miRgo^a^	0.8840	0.2900	0.7900	0.8760	0.1810	0.5905	0.7316
RNA22	0.3917	0.7593	0.4498	0.5453	0.1143	0.5571	0.5127
miRanda_0_0	0.0109	0.9959	0.1666	0.0216	0.0250	0.5125	0.0552
miRanda_0_C	0.4517	0.6141	0.4774	0.5927	0.0484	0.5242	0.5273
miRanda_S_0	0.0093	1.0000	0.1659	0.0185	0.0386	0.5193	0.0484
miRanda_S_C	0.4540	0.6058	0.4780	0.5943	0.0439	0.5220	0.5272
STMDB_3US	0.3419	0.7303	0.4033	0.4911	0.0560	0.5280	0.4680
STMDB_3ULS	0.6168	0.5394	0.6046	0.7243	0.1160	0.5580	0.6215
STMDB_CS	0.3084	0.7925	0.3849	0.4578	0.0809	0.5405	0.4523
STMDB_CLS	0.6589	0.5104	0.6354	0.7527	0.1280	0.5640	0.6417
STMDB_5US	0.0312	0.9834	0.1816	0.0602	0.0317	0.5159	0.1248
STMDB_5ULS	0.6098	0.4938	0.5915	0.7154	0.0769	0.5385	0.6066
TargetScan	0.5397	0.6307	0.5541	0.6709	0.1244	0.5622	0.5912
DIANA_microT	0.3076	0.7054	0.3705	0.4514	0.0103	0.5052	0.4352
PITA	0.0522	0.9876	0.2000	0.0990	0.0693	0.5346	0.1767
TarPmiR	0.7048	0.4896	0.6708	0.7829	0.1513	0.5757	0.6659
MBSTAR	0.3512	1.0000	0.4538	0.5199	0.2807	0.6404	0.5273
PACCMIT-CDS	0.0639	0.9461	0.2033	0.1189	0.0150	0.5075	0.1961

^a^miRgo, a abbreviation of miRgo_trA_FS-mRMR, was constructed by SVM with the mRMR feature selection method and trained on the trA training dataset.

### Performance evaluation with functionally important genes

**Figure 5:**
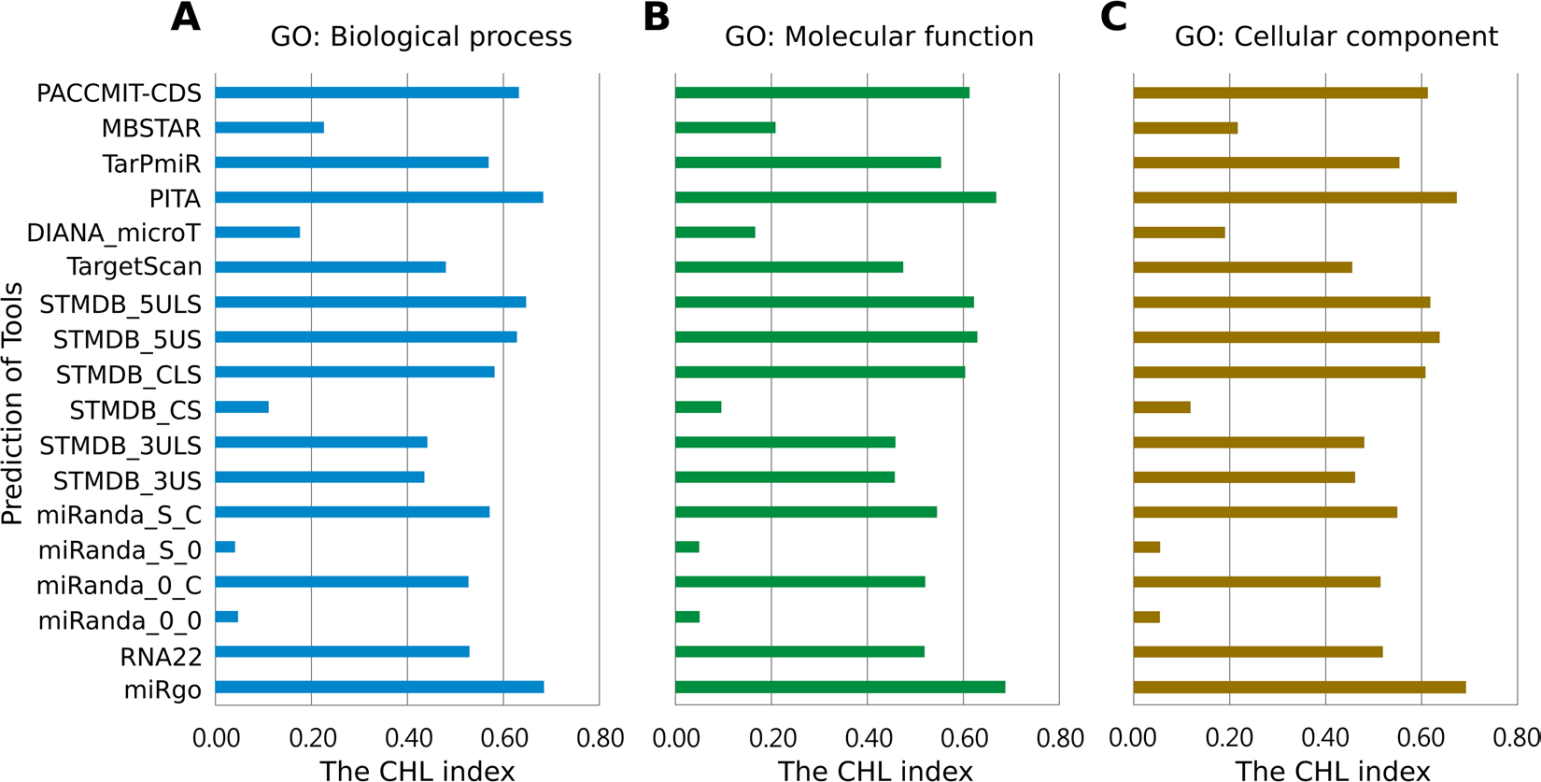
Comparison of the CHL Index of the different microRNA target site prediction methods on the functionally important gene sets. (**A**) For the genes with the biological process annotation. (**B**) For the genes with the molecular function annotation. (**C**) For the genes with the cellular component annotation.

The Gene Ontology resource (GO; http://geneontology.org) collects current scientific knowledge concerning the functions of genes and provides functional annotation of gene products^39^. All the knowledge regarding the functions of genes is supported by the scientific literature^43^. Therefore, genes with the GO annotation indicates that the functions of these genes have been investigated to some extent and imply that these genes might be interesting or functionally important. We are interested in the performance of miRgo in predicting the miRNA-target relationships of these genes with the GO annotation. The analysis was done by testing according to three types of functional data in the Gene Ontology database: biological process, molecular function, and cellular component. Independent testing data were categorized into three types, and all tools were tested based each category. miRgo performed better than all other tools in all three types of functional data, scoring 0.6841, 0.6899, and 0.6945 in biological process, molecular function, and cellular component, respectively, when evaluated by the CHL index. The results are shown in Fig. 5.

## Discussion

Prediction of miRNA–target relationships is important in biology because prediction of binding pairs may save time and material for experimental biologists. Here we described the integrated tool miRgo, which combines 11 features covering binding energy, scoring function, binding type, and sequence characteristics from six different prediction tools. The training set used for development, trA, was obtained by selecting the most-stable and least-stable binding pairs via an energy filter distribution function. The resulting classifiers showed high accuracy in prediction of both positive and negative data without overfitting. Compared with the integration of 11 tools, the integration of six tools and 11 features was superior in speed and accuracy.

Regarding miRNA–target interactions, the prediction of positive data is more important than that of negative data. To address this specific need, we developed a novel metric, the CHL index, which focuses more on Sp than the F1 score and focuses more on Sn than MCC. For example, STMDB_3US and MBSTAR have similar Sn values but very different Sp values (0.7303 and 1.0000) (Table 6). These two tools show a difference of 0.3 in the F1 score, but 0.6 in the CHL index, demonstrating that the CHL index is more Sp focused than is the F1 score. miRNADA_S_0 and MBSTAR have similar Sp values but very different Sn values (0.0093 and 0.3512). These two tools show a difference of 0.2421 for MCC but 0.4789 for the CHL index, demonstrating that the CHL index is more Sn focused than is MCC. Therefore the CHL index may have more discrimination power for examination of miRNA–target prediction models.

Compared with the previous prediction tools, miRgo was trained using the newest data from miRBase, containing 2,588 miRNAs. When tested with functional data from the Gene Ontology database and evaluated by the CHL index, it performed better than all other tools. A website (http://predictor.nchu.edu.tw/miRgo) has been built for users to assess miRgo. This tool takes gene name, gene ensemble ID, Refseq ID, gene sequence, and miRNA sequence as input and generates a prediction of binding status in addition to possible binding miRNAs.

## Supplementary information

Supplementary informations.
