# Supplementary material for: miRgo: integrating various off-the-shelf tools for identification of microRNA–target interactions by heterogeneous features and a novel evaluation indicator
Source: Sci Rep. 2020 Jan 30;10:1466. doi: 10.1038/s41598-020-58336-5 (PMC6992741; doi:10.1038/s41598-020-58336-5)
Supplement: Supplementary file 1 — Supplementary informations. [file 41598_2020_58336_MOESM1_ESM.pdf]

miRgo: integrating various off-the-shelf tools for identification of microRNA–target interactions by heterogeneous features and a novel evaluation indicator

Yen-Wei Chu[1,2,3,4,5,6], Kai-Po Chang[6,7], Chi-Wei Chen[1,8], Yu-Tai Liang[1], Zhi Thong Soh[1,9] and Li-Ching Hsieh[1,4,5,6,10,11]*

[1]Institute of Genomics and Bioinformatics, National Chung Hsing University, Taichung 402, Taiwan

[2]Agricultural Biotechnology Center, National Chung Hsing University, Taichung 402, Taiwan

[3]Institute of Molecular Biology, National Chung Hsing University, Taichung 402, Taiwan

[4]Biotechnology Center, National Chung Hsing University, Taichung 402, Taiwan

[5]Rong Hsing Research Center For Translational Medicine, National Chung Hsing University, Taichung 402, Taiwan

[6]Ph.D. Program in Medical Biotechnology, National Chung Hsing University, Taichung 402, Taiwan

[7]China Medical University Hospital, Taichung 404, Taiwan

[8]Department of Computer Science and Engineering, National Chung Hsing University, Taichung 402, Taiwan

[9]Department of Life Science, National Chung Hsing University, Taichung 402, Taiwan

[10]Advanced Plant Biotechnology Center, Biotechnology Center, National Chung Hsing University, Taichung 402, Taiwan

[11]Department of Physics, National Chung Hsing University, Taichung 402, Taiwan

*Correspondence and requests for materials should be addressed to L.-C.H. (email: liching@dragon.nchu.edu.tw)

Supplementary data

Table S1. The features encoded for each tool

Tool	Features [a]	Description of the features	Feature vector dimension
miRanda	mirSVR score (4)	an estimate of the miRNA effect on the mRNA expression level	48
	conservation (4)	a measurement of the conservation of nucleotide positions across multiple vertebrates	
	align score (4)	a score for each detected complementarity match between a miRNA and a potential target gene	
	energy (4)	the free energy of optimal strand-strand interaction between a miRNA and a potential target gene	
	gene start (4)	target gene start site	
	gene end (4)	target gene end site	
	microRNA start (4)	microRNA start site	
	microRNA end (4)	microRNA end site	
	gene ATCG (16)	the nucleotide composition of the binding region on the target	
TargetScan	seed type (7)	the type of a mature miRNA seed	14
	context score (2)	the sum of the contribution of a number of features for a specific binding site	
	Pct (1)	the probability of conserved targeting	
	position in the UTR (4)	miRNA binding position in the UTR	

RNA22	binding energy (1)	an assessment of how strong the binding is between the miRNA and its target mRNA	2
	p-value (1)	the likelihood that the target site loci is random	
	target sequence	target sequence	
	microRNA sequence	microRNA sequence	
RNAhybrid	minimum free energy (1)	the miRNA/mRNA duplex minimum free energy (MFE)	3
	p-value (1)	an assessment of the statistical significance of observed (normalized) MFEs	
	position (1)	position in target	
	target sequence	target sequence	
	microRNA sequence	microRNA sequence	
RNAduplex	binding site (4)	the miRNA binding site on the mRNA	5
	free energy (1)	the energy of duplex structure	
STarMirDB	site start (6)	start positions of the target region (site) predicted to be bound by miRNA	84
	site end (6)	end positions of the target region (site) predicted to be bound by miRNA	
	seed start (3)	start positions of the target sub-region complementary to the miRNA seed	
	seed end (3)	end positions of the target sub-region complementary to the miRNA seed	
	seed ATCG (12)	the nucleotide composition of a mature miRNA seed	

	seed type (21)	the type of a mature miRNA seed	
	site conservation (6)	conservation score by the PhastCons program for the binding site	
	seed conservation (3)	conservation score by the PhastCons program for the target sub-region complementary to the miRNA seed	
	ΔG_{hybrid} (6)	a measure of stability for miRNA:target hybrid as computed by RNAhybrid	
	ΔG_{nucl} (6)	a measure of the potential of nucleation for miRNA:target hybridization	
	ΔG_{total} (6)	a measure of the total energy change of the hybridization	
	logistic probability of the site (6)	probability of the site being an miRNA binding site as predicted by the logistic model in STarMirDB	
microT-CDS	miTG score (1)	a score for predicted interaction	1
PITA	position (1)	the miRNA binding site position	9
	seed type (2)	the type of a mature miRNA seed	
	ΔG_{duplex} (1)	the binding energy of the microRNA-target duplex	
	ΔG_{open} (1)	the free energy lost by unpairing the target-site nucleotides	
	$\Delta\Delta G$ (1)	an energy-based score for microRNA-target interactions	
	conservation (1)	site conservation	
	chromosome	the ID of the chromosome with the miRNA binding site	
	start (1)	the start position of the miRNA binding site	
	end (1)	the end position of the miRNA binding site	
PACCMIT-CDS	log(p-val) (1)	the observed number of conserved and/or accessible seed matches would appear in the target sequence by chance	2

	seed match positions (1)	the position of the sites complementary to the miRNA seed	
MBSTAR	seed type (7)	the type of a mature miRNA seed	10
	score(1)	a score for prediction confidence	
	binding site (2)	the miRNA binding site on the mRNA	
TarPmiR	binding probability (1)	the probability that the miRNA binds to the mRNA	6
	folding Energy (1)	the minimal folding energy calculated by RNAduplex	
	seed (1)	information about whether there existed a perfect pairing between 2-7 nt of miRNA	
	accessibility (1)	a measure of how likely a region in an target sequence is ‘open’ or accessible for an miRNA to bind	
	AU content (1)	AU-rich elements (AREs) are found of many mRNA, it was reported as an important feature for miRNA-mRNA binding	
	m/e motif (1)	an assessment of how different positions in miRNAs match the corresponding positions in target sites	
	Total features		184

[a]This column lists the name and dimension of the encoded feature

Table S2. Feature encoding of seed types

Seed types	Feature encoding code
offset 6mer site	0000001
6mer site	0000010
7mer-A1 site	0000100
7mer-m8 site	0001000
8mer site	0010000
3' compensatory site	0100000
Centered site	1000000

Table S3. Performance evaluation by various machine learning approaches

ALGORITHM	Sn	Sp	Acc	F_1-score	MCC	CHL-index
SVM	0.999	0.998	0.999	0.999	0.997	0.9986
PART	0.999	0.998	0.998	0.998	0.997	0.9985
HIRP	0.999	0.997	0.998	0.998	0.997	0.9983
HOEFFDINGTREE	0.998	0.998	0.998	0.998	0.997	0.9983
LOGISTIC	0.996	0.999	0.997	0.997	0.995	0.9975
MULTICLASSCLASSIFIER	0.996	0.999	0.997	0.997	0.995	0.9975
KSTAR	0.993	0.997	0.995	0.995	0.990	0.9950
BAYESNET	0.995	0.993	0.994	0.994	0.988	0.9940
A1DE	0.990	0.998	0.994	0.994	0.988	0.9938
IBK	0.990	0.996	0.993	0.993	0.986	0.9930
IBKLG	0.990	0.996	0.993	0.993	0.986	0.9930
NAIVEBAYES	1.000	0.956	0.978	0.978	0.957	0.9783
VOTEDPERCEPTRON	0.960	0.982	0.971	0.971	0.942	0.9710
RANDOMIZABLEFILTEREDCLASSIFIER	0.916	0.957	0.936	0.935	0.874	0.9361
ZEROR	0.800	0.200	0.500	0.615	0.001	0.5334
SGDTEXT	0.700	0.299	0.500	0.583	-0.001	0.5246
MULTISCHEME	0.800	0.199	0.500	0.615	-0.001	0.5329
STACKING	0.800	0.199	0.500	0.615	-0.001	0.5329
VOTE	0.800	0.199	0.500	0.615	-0.001	0.5329
INPUTMAPPEDCLASSIFIER	0.800	0.199	0.500	0.615	-0.001	0.5329

Table S4. Performance comparison of different miRNA–target interaction prediction methods for the trB set

Prediction method	Sn	Sp	Acc	F_1-score	MCC	MCC'	CHL-index
miRgo_trB[a]	0.9988	0.9921	0.9955	0.9955	0.9909	0.9955	0.9955
RNA22	0.5068	0.5487	0.5277	0.5176	0.0555	0.5277	0.5243
miRanda_0_0	0.1393	0.8953	0.5173	0.2239	0.0528	0.5264	0.3615
miRanda_0_C	0.1146	0.9579	0.5362	0.1981	0.1348	0.5674	0.3458
miRanda_S_0	0.1001	0.9484	0.5242	0.1738	0.0916	0.5458	0.3160
miRanda_S_C	0.0993	0.9791	0.5392	0.1773	0.1650	0.5825	0.3257
STMDB_3US	0.2562	1.0000	0.6281	0.4079	0.3833	0.6916	0.5465
STMDB_3ULS	0.4998	0.5267	0.5132	0.5066	0.0265	0.5132	0.5110
STMDB_CS	0.0000	1.0000	0.5000	null[b]	0.0000	0.5000	null[b]
STMDB_CLS	0.5325	0.4832	0.5078	0.5197	0.0157	0.5078	0.5117
STMDB_5US	0.0307	1.0000	0.5154	0.0596	0.1249	0.5624	0.1464
STMDB_5ULS	0.4826	0.5269	0.5047	0.4935	0.0095	0.5047	0.5009
TargetScan	0.8999	0.6451	0.7725	0.7982	0.5636	0.7818	0.7840
DIANA_microT	0.2388	0.9221	0.5805	0.3627	0.2204	0.6102	0.4903
PITA	0.0672	0.9476	0.5074	0.1201	0.0314	0.5157	0.2452
TarPmiR	0.7699	0.2454	0.5076	0.6099	0.0179	0.5090	0.5382
MBSTAR	0.3111	0.7481	0.5296	0.3980	0.0657	0.5329	0.4779
PACCMIT-CDS	0.0448	0.9882	0.5165	0.0848	0.0996	0.5498	0.1930

[a]The miRgo_TrB model was trained on the trB training data with 10-fold cross validation.

[b]null: Because Precision cannot be calculated in this case (division by zero), the F_1-score and the CHL-index cannot be calculated either.

Table S5. Performance comparison of different miRNA–target interaction prediction methods for the trC set

Prediction method	Sn	Sp	Acc	F_1-score	MCC	MCC'	CHL-index
miRgo_trC[a]	0.9515	0.8184	0.8850	0.8921	0.7768	0.8885	0.8885
RNA22	0.7577	0.8988	0.8282	0.8152	0.6631	0.8316	0.8249
miRanda_0_0	0.2005	0.9695	0.5850	0.3258	0.2659	0.6330	0.4718
miRanda_0_C	0.0558	0.9913	0.5236	0.1049	0.1334	0.5667	0.2271
miRanda_S_0	0.1192	0.9894	0.5543	0.2110	0.2204	0.6102	0.3667
miRanda_S_C	0.0466	0.9956	0.5211	0.0886	0.1336	0.5668	0.2004
STMDB_3US	0.2405	1.0000	0.6203	0.3878	0.3697	0.6849	0.5309
STMDB_3ULS	0.4548	0.7083	0.5815	0.5208	0.1686	0.5843	0.5606
STMDB_CS	0.0000	1.0000	0.5000	null[b]	0.0000	0.5000	null[b]
STMDB_CLS	0.4894	0.5075	0.4985	0.4939	-0.0031	0.4985	0.4969
STMDB_5US	0.0216	1.0000	0.5108	0.0424	0.1046	0.5523	0.1096
STMDB_5ULS	0.4498	0.5112	0.4805	0.4640	-0.0391	0.4804	0.4749
TargetScan	0.9656	0.9032	0.9344	0.9364	0.8705	0.9353	0.9354
DIANA_microT	0.2716	0.9969	0.6343	0.4262	0.3901	0.6950	0.5595
PITA	0.0925	0.9900	0.5412	0.1679	0.1870	0.5935	0.3161
TarPmiR	0.9616	0.8711	0.9163	0.9200	0.8361	0.9181	0.9181
MBSTAR	0.3022	0.9590	0.6306	0.4499	0.3464	0.6732	0.5667
PACCMIT-CDS	0.0759	0.9986	0.5373	0.1410	0.1935	0.5967	0.2822

[a]The miRgo_TrC model was trained on the trC training data with 10-fold cross validation.

[b]null: Because Precision cannot be calculated in this case (division by zero), the F_1-score and the CHL-index cannot be calculated either.

Table S6. Performance comparison of different miRNA–target interaction prediction methods for the trR set

Prediction method	Sn	Sp	Acc	F_1-score	MCC	MCC'	CHL-index
miRgo_trR[a]	0.9397	0.6600	0.7998	0.8244	0.6246	0.8123	0.8121
RNA22	0.4869	0.4225	0.4547	0.4717	-0.0908	0.4546	0.4602
miRanda_0_0	0.1604	0.8769	0.5186	0.2499	0.0535	0.5267	0.3832
miRanda_0_C	0.0437	0.9617	0.5027	0.0807	0.0136	0.5068	0.1835
miRanda_S_0	0.1217	0.9534	0.5376	0.2084	0.1354	0.5677	0.3563
miRanda_S_C	0.0419	0.9822	0.5121	0.0791	0.0710	0.5355	0.1823
STMDB_3US	0.2301	1.0000	0.6151	0.3741	0.3606	0.6803	0.5200
STMDB_3ULS	0.4575	0.5433	0.5004	0.4780	0.0008	0.5004	0.4927
STMDB_CS	0.0000	1.0000	0.5000	null[b]	0.0000	0.5000	null[b]
STMDB_CLS	0.4913	0.5023	0.4968	0.4940	-0.0064	0.4968	0.4959
STMDB_5US	0.0211	1.0000	0.5105	0.0412	0.1032	0.5516	0.1071
STMDB_5ULS	0.4776	0.5371	0.5073	0.4922	0.0147	0.5074	0.5022
TargetScan	0.9391	0.6289	0.7840	0.8130	0.5975	0.7987	0.7984
DIANA_microT	0.1967	0.9239	0.5603	0.3090	0.1756	0.5878	0.4463
PITA	0.0790	0.9465	0.5128	0.1395	0.0513	0.5256	0.2723
TarPmiR	0.7778	0.1764	0.4771	0.5980	-0.0573	0.4713	0.5094
MBSTAR	0.2827	0.7475	0.5151	0.3682	0.0340	0.5170	0.4551
PACCMIT-CDS	0.0456	0.9882	0.5169	0.0862	0.1013	0.5506	0.1955

[a]The miRgo_TrR model was trained on the trR training data with 10-fold cross validation.

[b]null: Because Precision cannot be calculated in this case (division by zero), the F1-score and the CHL-index cannot be calculated either.

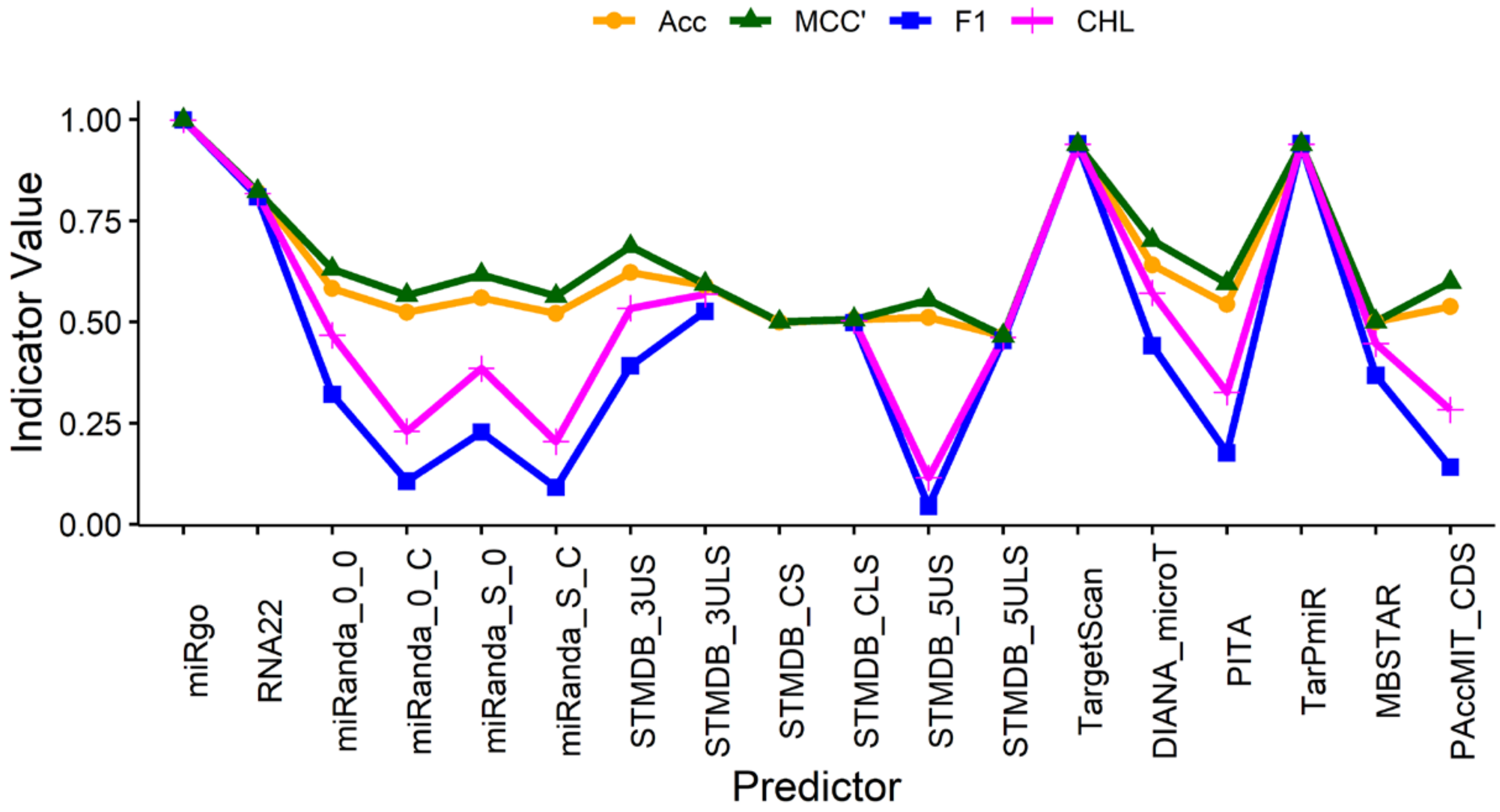

Figure S1. Correlation between four indicators, Acc, the F1 score, MCC' and the CHL index score, measured by testing with the trA set and various predictors. The F1-score and the CHL-index cannot be calculated for STMDB_CS because both TP and FP are zeros in this case.
